# Supplementary material for: High-resolution single-particle imaging at 100–200 keV with the Gatan Alpine direct electron detector
Source: J Struct Biol. Author manuscript; Available in PMC 2024 Nov 7. (PMC11542591; doi:10.1016/j.jsb.2024.108108)

SUPPLEMENTAL INFORMATION

Table S1. Data collection parameters for each collection

	Magnification (nominal/ calibrated)	Total dose ($e^-/Å^2$)	Total exposure (s)	Exposure			Dose rate (e^-/pix/s)	Mode	Pixel size (Å/phys pix)	MotionCor2 (v1.4.0)
Alpine 120 keV TFS Glacios	43,000 / 58,823	58.9	4.95	0.595 e^-/A^2/frame	0.05 s/frame	99 frames	8.6	CDS	0.85	3x4 patches Group 3 frames
Alpine 200 keV TFS Glacios	43,000 / 54,229	45.5	4.95	0.46 e^-/A^2/frame	0.05 s/frame	99 frames	7.8	CDS	0.922	3x4 patches Group 3 frames
K3 200 keV TFS Glacios	54,000 / 68,493	55.1	1.84	0.60 $e^-/Å^2$/frame	0.02 s/frame	92 frames	16	nonCDS	0.73	7x5 patches Group 1 frame
K3 300 keV Titan Krios (LKB1)	105,000 / 59,880	45.8	2.01	0.57 $e^-/Å^2$/frame	0.025 s/frame	80 frames	16	nonCDS	0.835	7x5 patches Group 1 frame
K3 300 keV Titan Krios (apoferritin)	105,000 / 59,880	47.7	2.00	0.596 $e^-/Å^2$/frame	0.025 s/frame	80 frames	16	nonCDS	0.8189	7x5 patches Group 1 frame
Alpine 200 keV TFS Talos F200C (apoferritin)	45,000 / 57,339	51.4	5.04	1.02 $e^-/Å^2$/frame	0.1 s/frame	50 frames	7.8	CDS	0.872	3x4 patches Group 1 frames
Alpine 100 keV TFS Talos F200C (apoferritin)	45,000 / 58,685	143.3	19.90	0.72 $e^-/Å^2$/frame	0.1 s/frame	199 frames	5.2	CDS	0.852	3x4 patches Group 1 frames
Alpine 100 keV TFS Talos F200C (aldolase)	45,000 / 58,685	71.6	9.94	0.72 $e^-/Å^2$/frame	0.1 s/frame	99 frames	5.2	CDS	0.852	3x4 patches Group 1 frames
Alpine 100 keV TFS Talos F200C (transthyretin)	45,000 / 58,685	50.1	6.96	0.72 $e^-/Å^2$/frame	0.1 s/frame	70 frames	5.2	CDS	0.852	3x4 patches Group 1 frames

Table S2. Comparison of image processing and reconstructions for apoferritin TFS Glacios and Titan Krios collections

	Initial micrographs (no.)	Curated micrographs (no.)	Initial particles (no.)	Final particles (no.)	NU-Refinement	
					Res (Å)	B-factor
Alpine 120 keV TFS Glacios	3,789	3,312	1,635,971	200,000	1.92	65.2
Alpine 200 keV TFS Glacios	5,046	3,619	1,834,404	204,387	1.76	57.7
K3 200 keV TFS Glacios	5,097	3,903	2,675,691	200,000	1.98	79.7
K3 300 keV Titan Krios	622	617	790,751	200,000	1.82	65.4

Table S3. Comparison of image processing and reconstructions for LKB1 complex TFS Glacios and Titan Krios collections

	Initial micrographs (no.)	Curated micrographs (no.)	Initial particles (no.)	Final particles (no.)	NU-Refinement	
					Res (Å)	B-factor
Alpine 120 keV TFS Glacios	3,635	2,868	897,749	120,684	3.19	152.3
Alpine 200 keV TFS Glacios	3,635	3,179	1,229,097	111,117	3.37	163.2
K3 200 keV TFS Glacios	1,504	1,337	1,000,313	144,801	3.37	177.1
K3 300 keV Titan Krios	1,293	1,223	979,927	124,780	2.86	121.1

Table S4. Comparison of image processing and reconstructions for TFS Talos F200C collections

	Scope/ detector setup	Initial micrographs (no.)	Curated micrographs (no.)	Initial particles (no.)	Final particles (no.)	NU-Refinement	
						Res (Å)	B-factor
apoferritin	Alpine 100 keV Talos	310	218	82,977	59,000	2.60	103.4
	Alpine 200 keV Talos	100	84	29,404	16,000	2.70	90.9
aldolase	Alpine 100 keV Talos	2,476	2,027	1,914,721	674,124	3.09	189.2

Table S5. Details on the Gatan Alpine camera

	Internal frame rate	Sensor size	Physical pixel	Frame rate available to user
Gatan Alpine	1502 fps	2,304 x 3,240 pixels	5 μm	75 fps

Table S6. Statistics of data collection, cryo-EM refinement, and the resulting atomic model for LKB1 complex collected on TFS Titan Krios with Gatan K3

LKB1 complex	
Data collection and processing	
Magnification	105,000
Voltage (kV)	300
Electron exposure (e^-/Å^2)	69
Defocus range (μm)	-1.0 to -2.0
Physical pixel size (Å)	0.835
Map resolution (Å)	2.86 (FSC 0.143)
Model Refinement	
Initial model used (PDB code)	2WTK
Model resolution (Å)<br>(FSC threshold 0.143/0.5)<br>CC volume/mask	 2.8/3.0 (0.143/0.5)<br>0.82/0.85
Model composition<br>Atoms non-H and H<br>Protein residues<br>Ligands	 15249 (H:7652)<br>941<br>ADP: 1
B factors (Å^2) min/max/mean<br>Protein<br>Ligand	 17.37/95.49/38.26<br>31.67
R.m.s. deviations<br>Bond lengths (Å)<br>Bond angles (°)	 0.013<br>1.764
Validation<br>MolProbity score<br>Clashscore<br>Poor rotamers (%)<br>CaBLAM outliers (%)<br>Q-score	 0.53<br>0.07<br>0.12<br>0.97<br>0.68
Ramachandran plot<br>Favored (%)<br>Allowed (%)<br>Disallowed (%)	 98.18<br>1.82<br>0.00
Data Availability	
EMDB<br>PDB	EMD-43506<br>PDB: 8VSU

Supplemental Figure 1: Increased reliability and repeatability of DQE measurements. (A) An illustration of the Gatan DQE module, which houses two retractable knife edges and a retractable Faraday cup. The module is positioned directly above the camera housing. Specially prepared and coated knife edge blades were employed to enhance the precision and consistency of DQE measurements. Representative images of the centered half-plane horizontal and vertical edge used for DQE measurements are shown in B and C, respectively. 200 keV edge DQE measurements at 7.5 electrons pixel^{-1} second^{-1} from nine sensors (D) and edge DQE measurements on two different sensors at 300 keV show consistent and reproducible results (E).

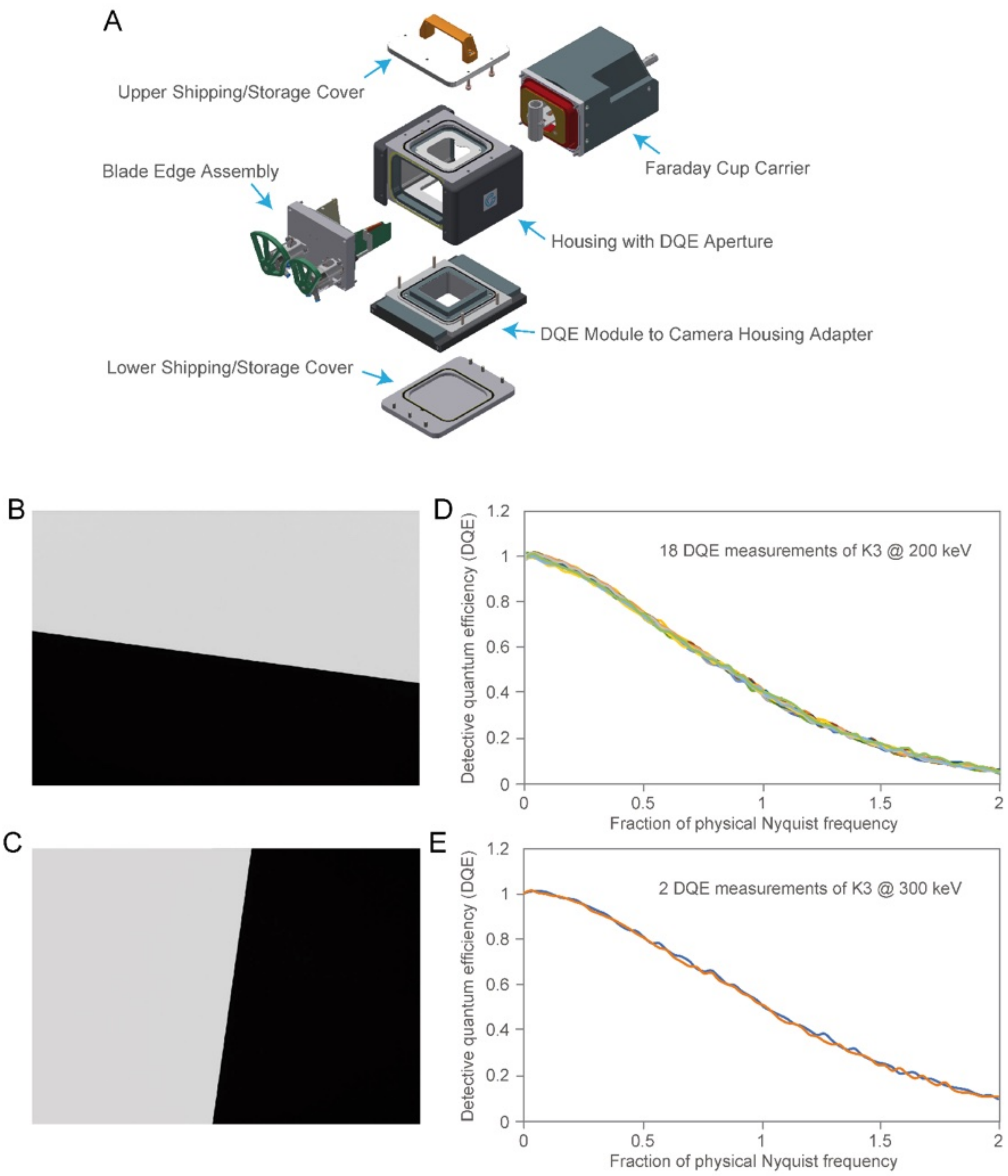

Supplemental Figure 2: Sparse 100 keV electron events captured by K3 and Alpine cameras. Enhanced detection of single 100 keV electron events is achieved by optimizing the detection layer and surrounding pixel design. The Alpine camera (B) exhibits superior sharpness of the incident electrons compared to the K3 camera (A).

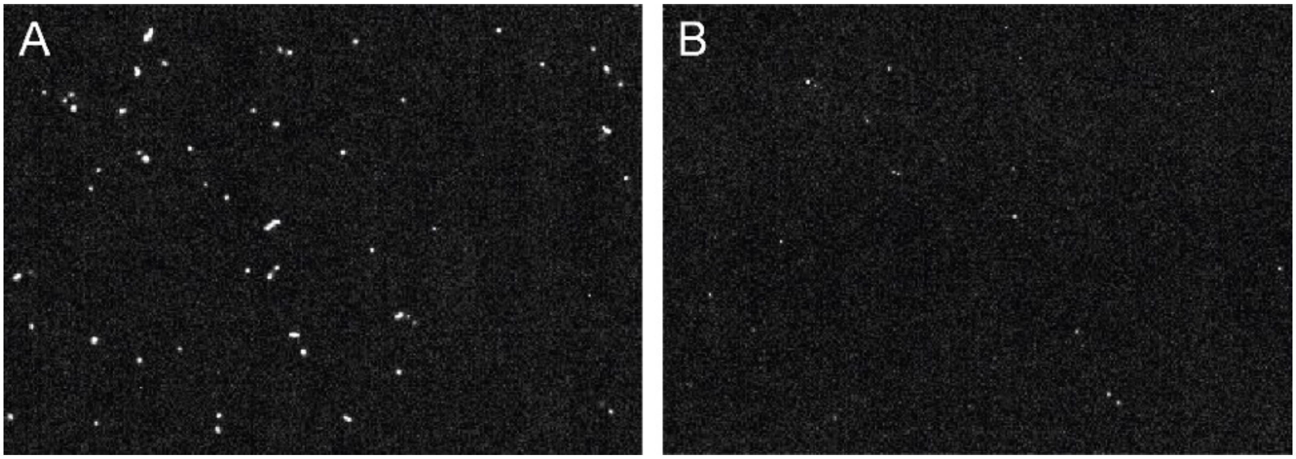

Supplemental Figure 3: Noise power spectra (NPS) and modulation transfer function (MTF) of Alpine at 100 and 200 keV and K3 at 200 keV. The Alpine camera demonstrates a response that closely matches that of the K3 camera at 200 keV, indicating comparable performance. (A) NPS and (B) MTF were measured in super-resolution counting CDS mode at 7.5 electrons pixel^{-1} second^{-1}.

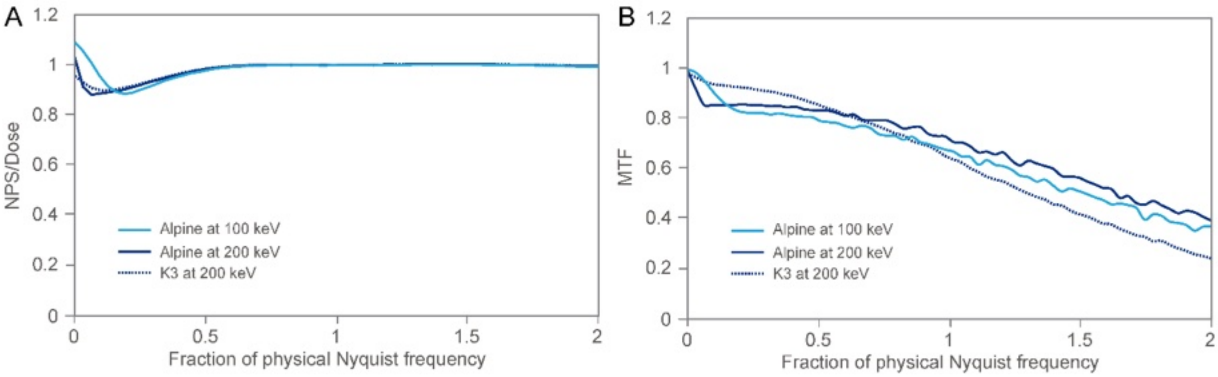

Supplemental Figure 4: Gold-standard and directional FSC curves for the apoferritin reconstructions from data collections collected with the Alpine detector with the TFS Glacios at (A) 120 keV and (B) 200 keV, and the K3 detector on the (C) TFS Glacios at 200 keV and the (D) TFS Titan Krios at 300 keV.

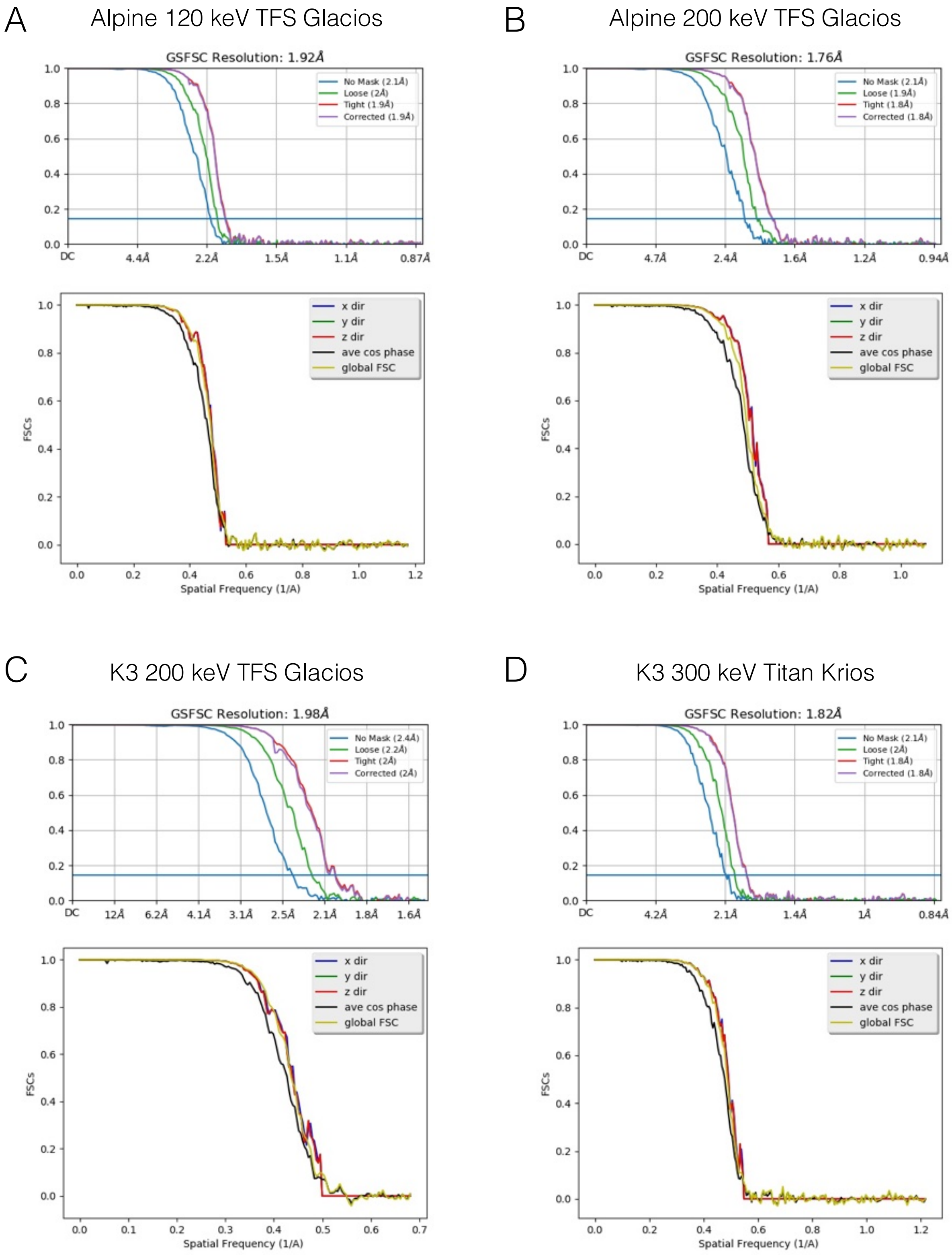

Supplemental Figure 5: The LKB1 complex imaging and reconstruction data for Alpine detector at 120 keV and 200 keV. (A) Schematic of LKB1 heterocomplex and subcomplex. LKB1 shown in pink, MO25α shown in blue, and STRADα shown in green. Representative micrograph, 2D class averages, gold-standard FSC curves, and directional FSC curves for all data collections collected with the Alpine detector with the TFS Glacios at (B) 120 keV and (C) 200 keV. Novel 2D class average for subcomplex is demarcated by purple border.

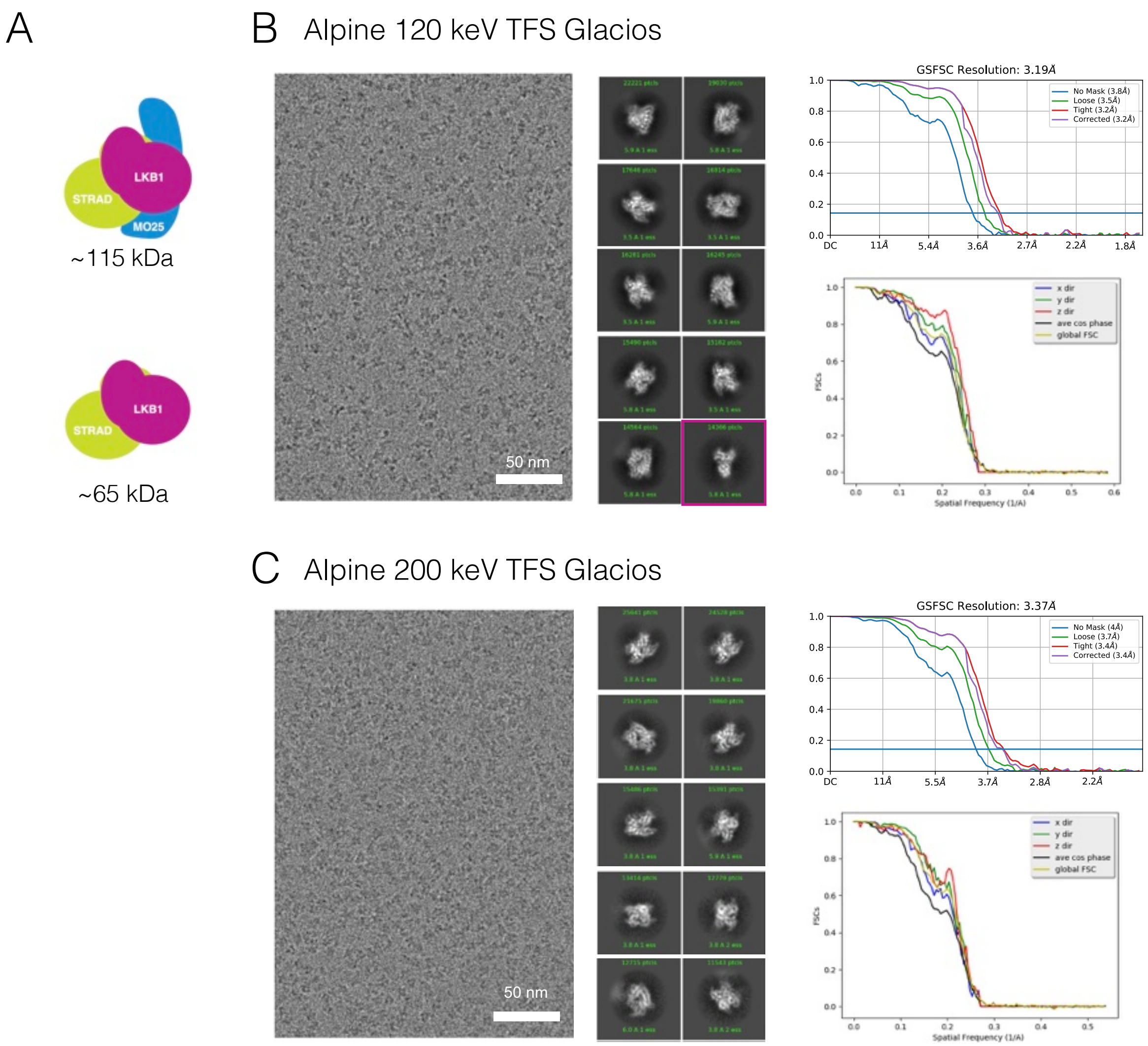

Supplemental Figure 6: The LKB1 complex imaging and reconstruction for K3 detector collections. Representative micrograph, 2D class averages, gold-standard FSC curves, and directional FSC curves for data collections with the K3 detector on the (A) TFS Glacios at 200 keV and the (B) TFS Titan Krios at 300 keV.

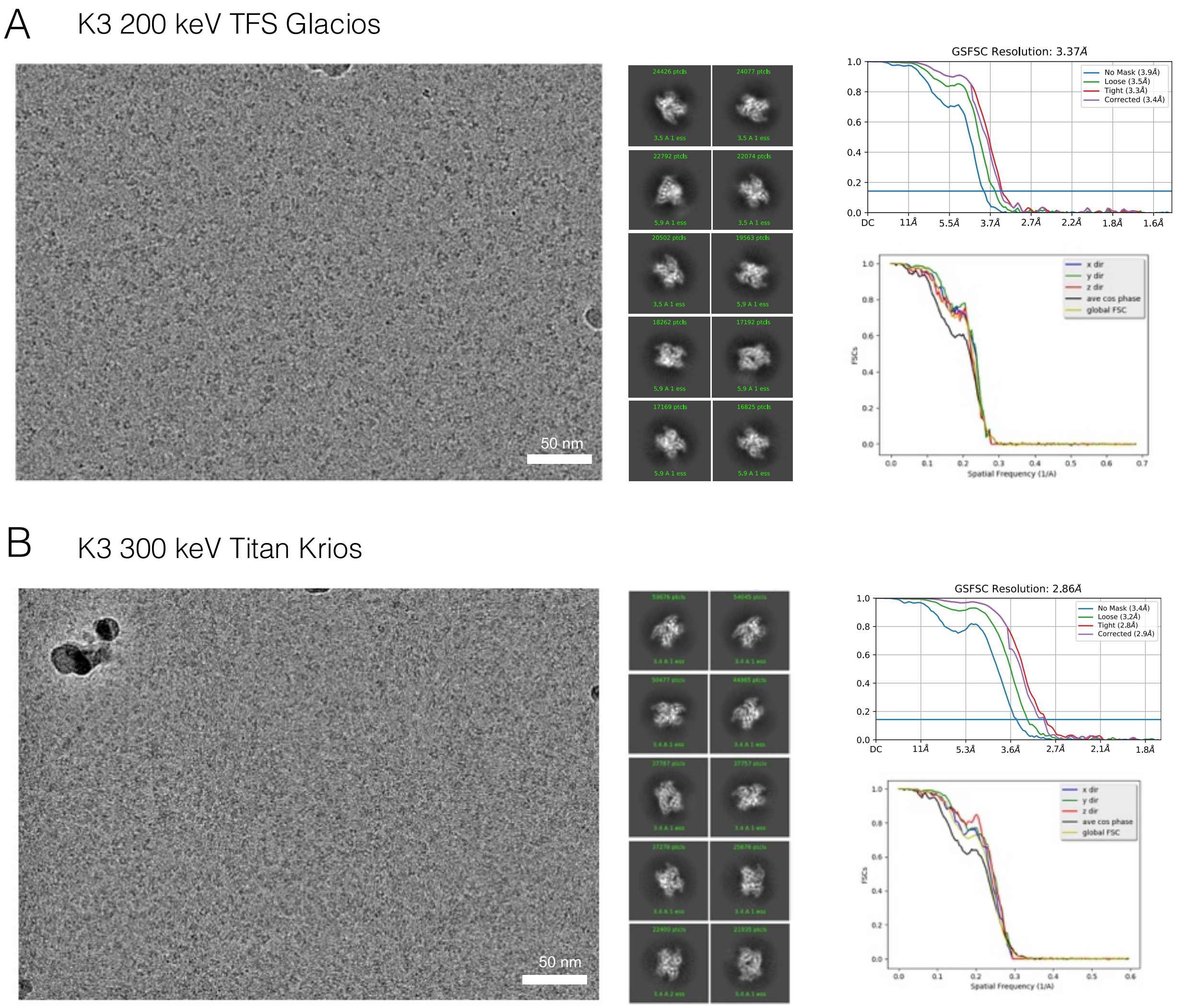

Supplemental Figure 7: LKB1-STRADα-MO25α heterocomplex is in a different nucleotide state in cryo-EM reconstruction as compared to the previously published crystal structure. A) Cryo-EM map and the resulting model of the LKB1 heterocomplex with LKB1 shown in pink, MO25α shown in blue, and STRADα shown in green. B) Overlay of the cryo-EM built model in ribbon (colored) and the crystal structure of the LKB1 complex (PDB: 2WTK) in grey. C) Zoomed-in view showing cryo-EM density of the nucleotide pocket of the ADP-bound STRADα pseudokinase. D) Zoomed-in view showing cryo-EM density of the nucleotide pocket of the apo LKB1 kinase. AMPPNP from the crystal structure is shown to demonstrate lack of its cryo-EM density.

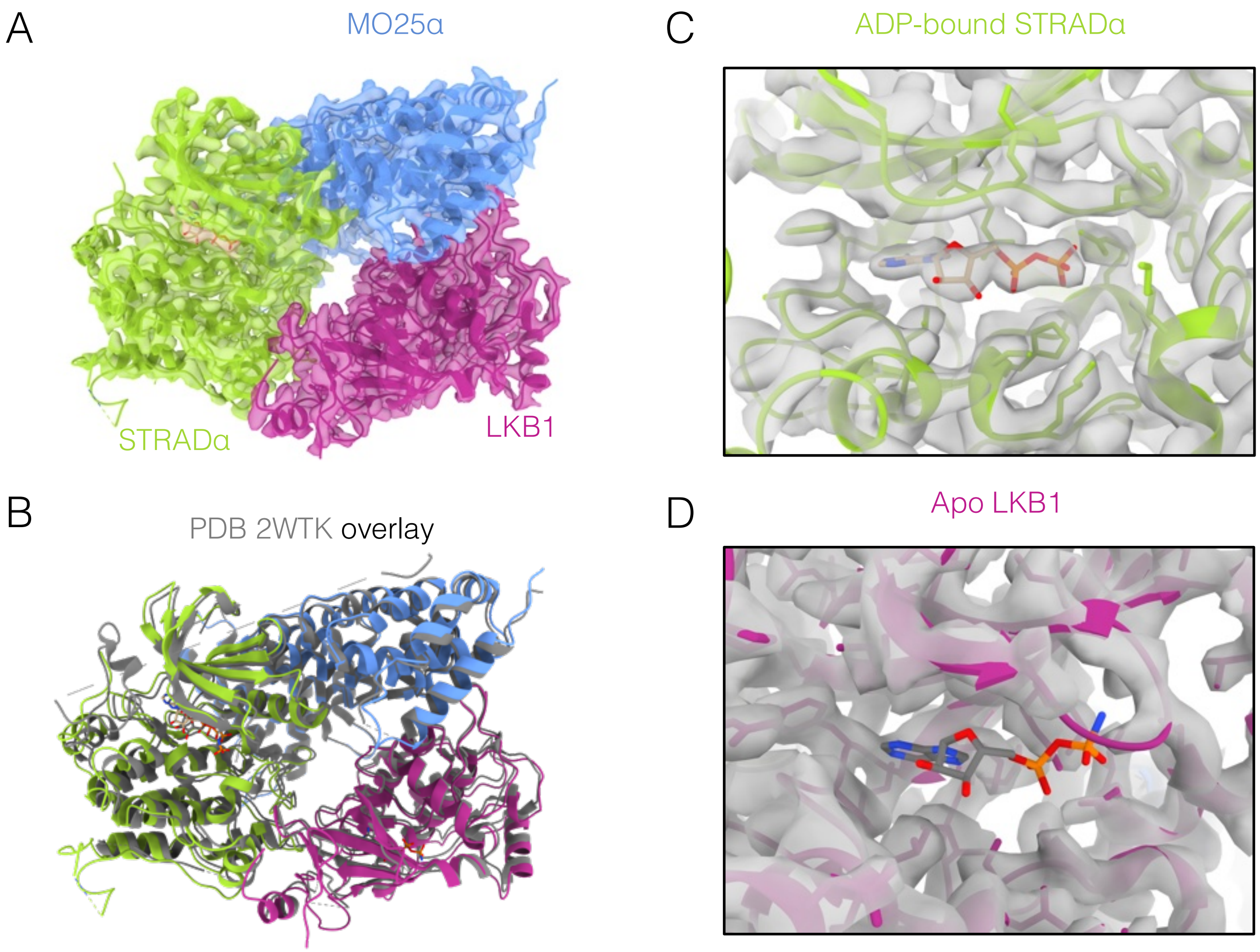

Supplemental Figure 8: Notable differences between cryo-EM model and the crystal structure of LKB1-STRADα-MO25α heterocomplex. A) Cryo-EM density and resulting model for MO25α (blue ribbon) with overlay of the STRADα WEF motif from the crystal structure in green showing no density for that motif. Two rotamer conformations of Mo25α ARG256 with EM density in light blue. B) Cryo-EM map (transparent gray) and model colored by protein (as in sup fig 4A). C terminal region of STRADα for which cryo-EM density is missing is outlined in red that and is at the crystal packing interface with the neighboring molecule shown in gray surface. C) Now resolved LKB1 C-terminal Flanking Tail (pink) overlayed with the crystal structure (gray). Dashed lines represent missing residues. Novel interactions at binding interfaces between LKB1 C-terminal tail and STRADα at (D) αG helix and (E) αEF-αF loop are displayed. D) Additional residues observed in the cryo-EM structure are shown in light pink. Interactions shown by dotted lines. E) EM density and fitted model of the C-terminal flanking tail interaction of TRP332 with GLN254 of the STRADα αEF-αF loop.

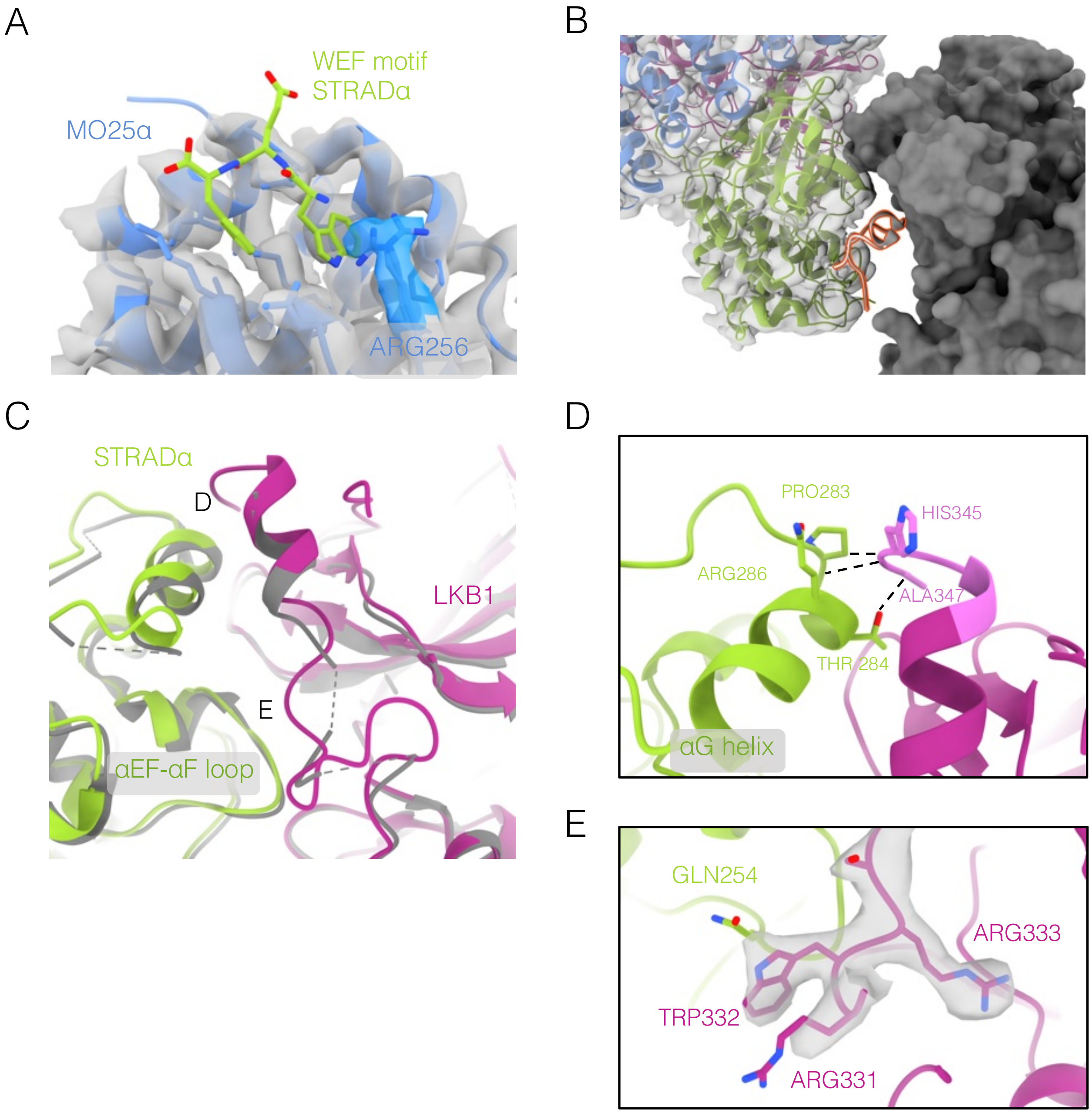

Supplemental Figure 9: Comparison of image quality using per-particle SSNR in terms of fraction of Nyquist. A plot of ppSSNR as a function of fraction of physical Nyquist for (A) apoferritin and (B) LKB1 collected using the Alpine detector at 120 keV (blue), 200 keV (red), and the K3 detector at 200 keV (green) and 300 keV (yellow). See Table S1 for explicit pixel sizes associated with these collections.

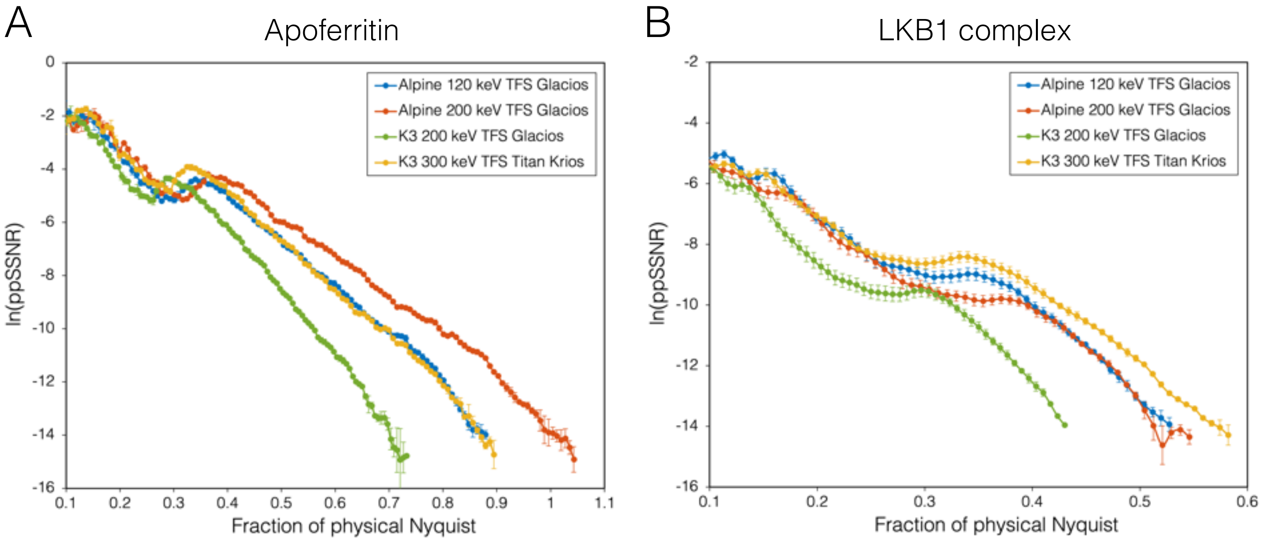

Supplemental Figure 10: 200 keV apoferritin reconstruction using the Alpine detector on a Talos F200C microscope with a side-entry cryo-holder. 3D reconstruction of apoferritin colored by local resolution (left), and the gold-standard global FSC curve and the directional FSC curves (right).

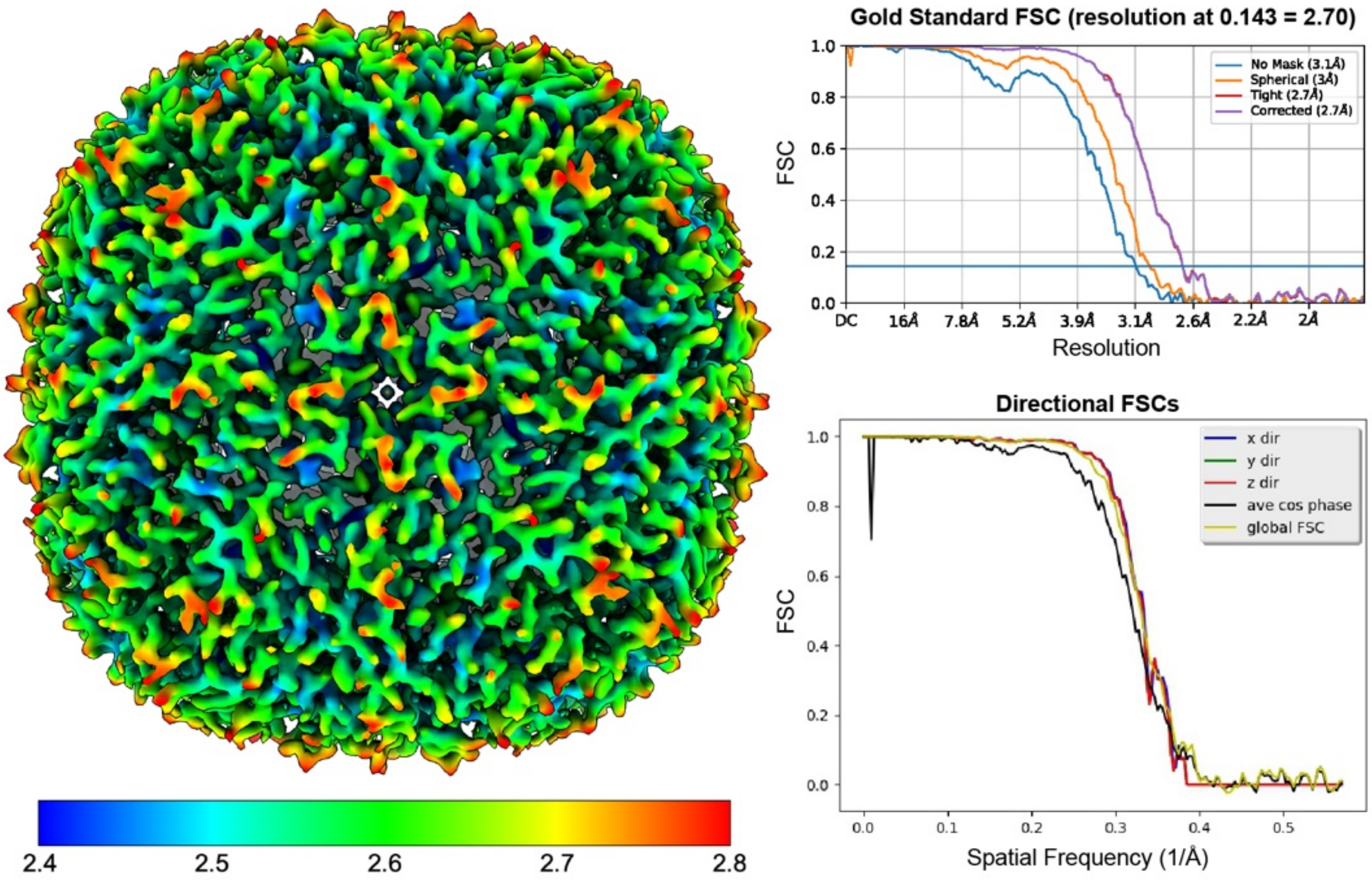

Supplemental Figure 11: 100 keV imaging and reconstruction with the Alpine detector on a Talos F200C with a side-entry cryo-holder. Representative micrograph, 2D class averages, gold-standard FSC curves, and directional FSC curves for (A) apoferritin and (B) aldolase samples.

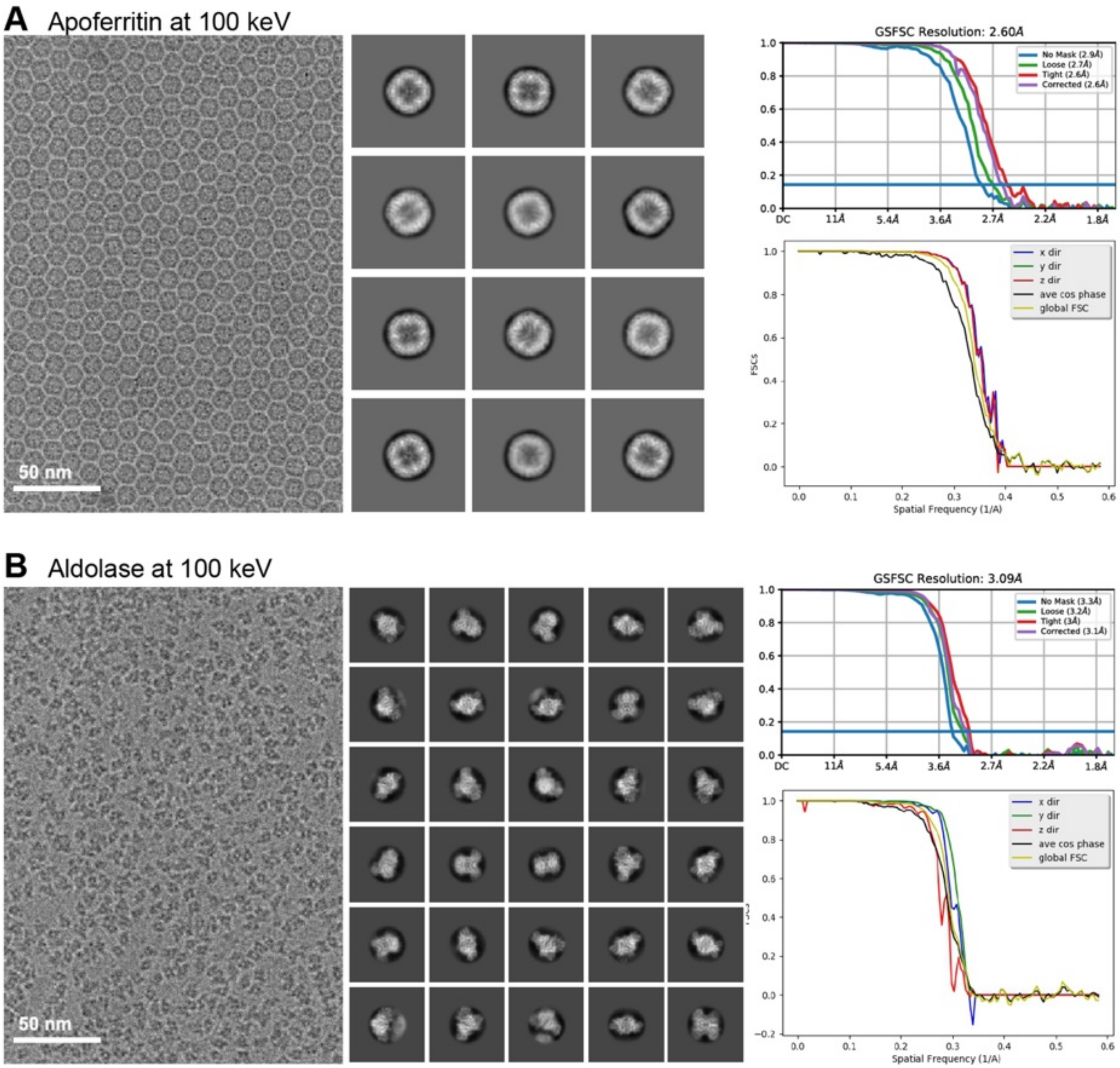

Supplemental Figure 12: 100 keV imaging of transthyretin (55 kDa) on graphene. Representative micrograph (left) and 2D class averages (right) of transthyretin sample imaged with the Alpine detector on a Talos F200C at 100 keV with a side-entry cryo-holder.

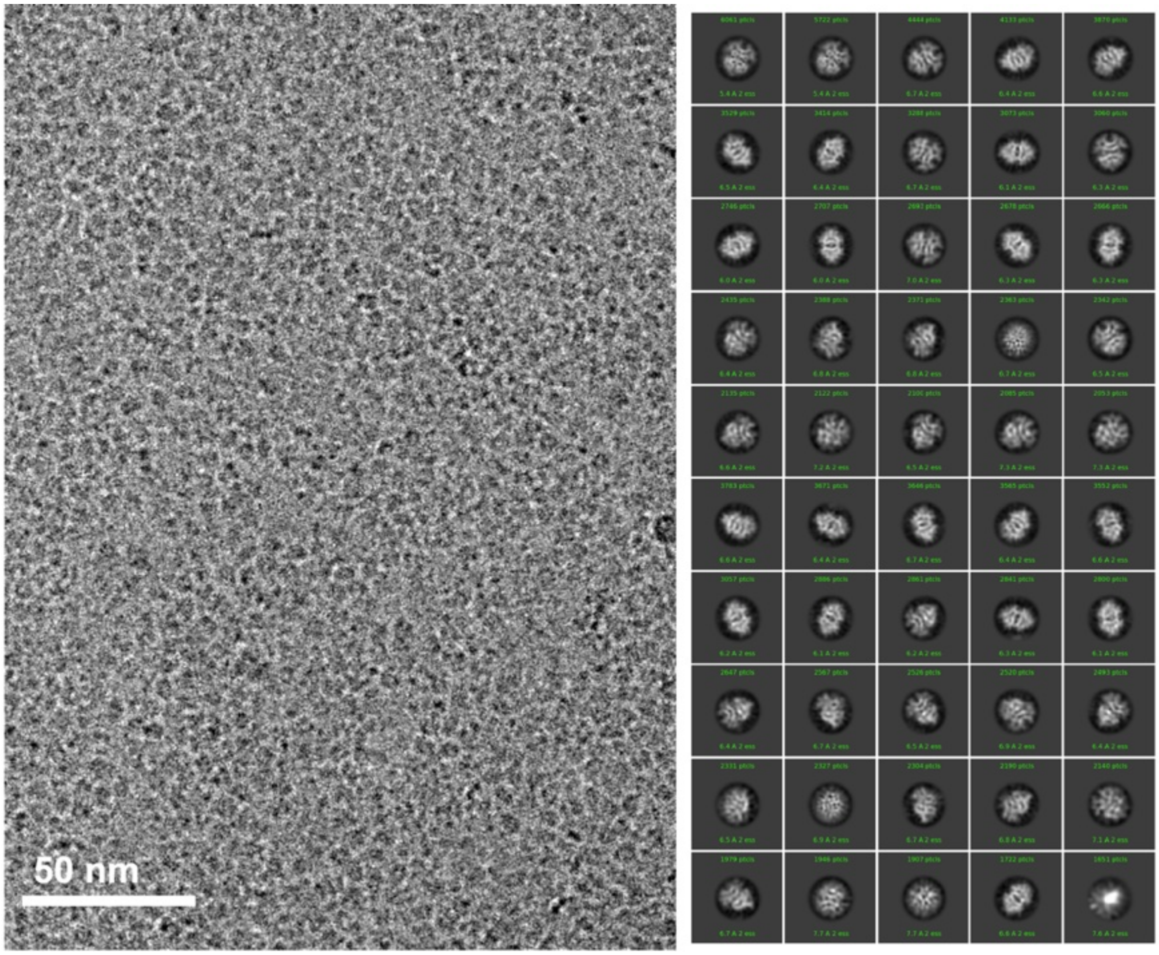

Supplement: supplementary material [file NIHMS2029523-supplement-supplementary_material.pdf]
